# Defining a new vision for the retinoblastoma gene: report from the 3rd International Rb Meeting

**DOI:** 10.1186/1747-1028-8-13

**Published:** 2013-11-21

**Authors:** Seth M Rubin, Julien Sage

**Affiliations:** 1 Department of Chemistry and Biochemistry, University of California, Santa Cruz, CA 95064, USA; 2 Departments of Pediatrics and Genetics, Stanford University, Stanford, CA 94305, USA

**Keywords:** Retinoblastoma, Rb, p107, p130, E2F, CDK, Cyclin

## Abstract

The retinoblastoma tumor suppressor (Rb) pathway is mutated in most, if not all human tumors. In the G0/G1 phase, Rb and its family members p107 and p130 inhibit the E2F family of transcription factors. In response to mitogenic signals, Cyclin-dependent kinases (CDKs) phosphorylate Rb family members, which results in the disruption of complexes between Rb and E2F family members and in the transcription of genes essential for S phase progression. Beyond this role in early cell cycle decisions, Rb family members regulate DNA replication and mitosis, chromatin structure, metabolism, cellular differentiation, and cell death. While the RB pathway has been extensively studied in the past three decades, new investigations continue to provide novel insights into basic mechanisms of cancer development and, beyond cancer, help better understand fundamental cellular processes, from plants to mammals. This meeting report summarizes research presented at the recently held 3rd International Rb Meeting.

## Background

The Rb tumor suppressor was cloned more than 25 years ago from children with retinoblastoma [1-4]. This seminal discovery led to an intense research effort culminating in the elucidation of the Rb pathway and fundamental mechanisms governing the G1/S transition of the cell cycle. It is now understood that regulators and mediators of Rb function are deregulated in a large set of diverse pediatric and adult tumors. In the last 10 years, a number of experiments have shown that Rb controls many biological processes beyond cell cycle entry, including at other stages of the cell cycle, for cell survival and during cellular differentiation. At the molecular level, while E2F transcription factors are known to be critical mediators of Rb function, the Rb protein binds to more than 150 other proteins, such as tissue-specific transcription factors and chromatin remodeling enzymes (see [5-9] for recent reviews).

Major challenges in the field include determining the biochemical mechanisms carried out by multiple Rb-containing complexes in cells, exploring the role of novel Rb functions in tumor suppression, and identifying the combinations of genetic alterations that result in tissue-specific cancers. The ultimate goal of the field is to discover novel therapeutic approaches to stop or slow the growth of human tumor cells with mutations in the Rb pathway. Accordingly, research on Rb and the networks around Rb in cells remains intense with publication of nearly 1,000 relevant journal articles a year.

Two previous international Rb conferences were organized in 2009 and 2011 in Toronto, Canada by Eldad Zacksenhaus and Rod Bremner. The success of these first two meetings coalesced a large group of investigators with a strong interest in participating in a scientific meeting focusing on the Rb pathway, which would be organized every other year in a rotating manner by active participants. 88 researchers in the Rb field recently gathered to exchange results and ideas at the 3rd International Rb Meeting, which was held October 7–10, 2013, in Monterey, CA, USA. The conference included 33 oral presentations and 45 posters. While we cannot summarize here all these studies, many of them unpublished, we highlight several topics discussed.

## Meeting summary

A number of presentations focused on the disease of retinoblastoma, the pediatric tumor after which the Rb gene was named. The recent publication of the first human retinoblastoma cancer genomes by Michael Dyer’s group emphasized the very low number of alterations found in these tumors and suggested an epigenetic mechanism of tumorigenesis upon loss of Rb function [10-12]. Claudia Benavente from the Dyer lab presented new analyses of the genomes of pediatric tumors, including cancers with *RB* mutations, and the St Jude’s Children’s Research Hospital now provides access to a large number of data and reagents (https://hospital.stjude.org/dbstp/). A number of other groups, including those of Josephine Dorsman and David MacPherson, are performing genomics studies on patient-derived retinoblastomas as well as tumors from genetically engineered mice [13-18]. While some of the human tumors clearly develop with few DNA alterations beyond Rb loss, these alterations may still provide key insights into the mechanisms of tumorigenesis upon loss of Rb function. Genomics and epigenomics studies of retinoblastoma and other Rb-deficient tumors are still in their infancy and, combined with cellular systems and mouse models, may identify novel therapeutic targets. In stimulating new work that could complement mouse models, David Cobrinik and his colleagues are exploring the mechanisms of cancer initiation in human fetal retinal cells upon Rb loss [19].

While Rb was identified nearly three decades ago, there are still no targeted therapies to treat Rb-deficient tumors. In an exciting development, several presenters discussed remarkable progress towards developing such therapeutics. Work from the laboratory of Erik Knudsen has underscored the differential response of Rb wild-type and Rb-deficient breast cancer cells to chemotherapy, the latter often being more sensitive to classical chemotherapeutic agents [20,21]. Recent results from the laboratory of Rod Bremner demonstrate that reducing E2F or Cdk2 activity using small molecule inhibitors, even for a short period of time early during tumor development in mice, may be sufficient to prevent the growth of retinoblastoma [22]. These experiments and ongoing work suggest that such “prevention” strategies may help significantly reduce tumor burden in familial cases or when tumors are detected early. Beyond this targeted approach, other groups, including those of Eldad Zacksenhaus and Maria Alvarado-Kristensson, are performing high throughput screens to identify small molecules that may specifically block the expansion of Rb mutant cells, including Rb-deficient triple negative breast cancer [23].

One of the most interesting aspects of the conference was the large number of presentations introducing novel functions for Rb pathway members. The groups of Peter Sicinski, Philip Hinds, and Philipp Kaldis all identified novel functions for Cyclins and CDKs using state-of-the-art mouse genetics approaches. These functions go beyond the classical cell cycle progression roles for these kinase complexes, and extend to the control of differentiation and organ/tissue function [24]. Similarly, the groups of Nicholas Dyson, Maxim Frolov, William Henry, David Johnson, Jacqueline Lees, and Chiaki Takahashi found new roles for Rb and E2F in various central cellular processes, including mitochondrial function, metabolism, the transcription of small RNAs, RNA translation, DNA repair, or cell migration [7,25,26]. Work from the laboratories of Timothy Hallstrom, Gustavo Leone, James Pipas (with Maria Teresa Saenz Robles), Julien Sage, and Ruth Slack underscored functional interactions between E2F transcription factors and other transcription factors such as beta-catenin, Sox2, Myc, YAP, or FoxO, uncovering complex regulatory networks controlling multiple cellular processes (e.g. [27-31]). The number of partners for Rb and E2F family members and the multitude of functions that they exert in cells bring the field to a new level of complexity.

A number of groups, including the laboratories of Ashby Morrison, Elizaveta Benevolenskaya, Jesus Paramio, and Fred Dick presented new evidence of a role for Rb in regulating chromatin structure using a combination of biochemical, molecular, and genetic studies [32,33]. Several groups (Seth Rubin, Joe Lipsick, James DeCaprio, Valerie Reinke, Susan Strome) have begun to explore the mechanisms of action of the DREAM (DP, Rb, E2F, and MuvB) and Myb-MuvB complexes in cells, including the identity and the structure of these complexes, how they control gene expression during the cell cycle and development, and how the complexes are regulated [34-36].

Another new area of investigation described at the conference was the analysis of cell cycle progression in single cells by Jan Skotheim, Lingchong You, and Tobias Meyer labs (postdoctoral fellow Sabrina Spencer) (e.g. [37-39]). When presented next to new results from the laboratory of Steven Dowdy (by Manuel Kaulich) on the kinetics of Rb phosphorylation by CDKs, these experiments help redefine the restriction point and when cells are committed to enter and conclude a cell cycle. Together these studies may soon modify the old textbook view of the G1/S checkpoint and the role of CDK activity in defining this checkpoint.

The Rb field has been primarily driven by the role of the Rb pathway in cell cycle control and cancer. However, interesting work in yeast (Jan Skotheim), in *C. elegans* (Susan Strome, Valerie Reinke), in *D. melanogaster* (Maxim Frolov, Nicholas Dyson, Joe Lipsick), and in plants (Wilhelm Gruissem and Arp Schnittger) was presented on the role of Rb-like and E2F-like molecules [35,40-47]. These studies further highlight a role of Rb in cell fate decisions that may have been conserved during evolution from fungi to mammals and plants [48].

## Conclusions

The Rb field is vibrant and relevant to many areas of biology, including cancer biology, developmental biology, stem cell biology, and regenerative medicine. A major goal of the Rb meeting, and its highest impact, is to offer a unique forum for building a community of scientists working together, advancing scientific knowledge. The 3rd International Rb Meeting offered hope that 20 years of molecular studies would soon translate into novel therapeutic options in a large number of patients. At the same time, the conference further highlighted the need for many more years of biochemical, structural, cellular, and organismal studies to better understand the regulation and the mode of action of Rb in plants and animals. The 4th International Rb Meeting, which will take place in Boston in 2015 and will be organized by Drs. J. Lees (MIT), N. Dyson (MGH, Harvard Medical School), and J. DeCaprio (DFCI, Harvard Medical School), will with no doubt reveal further unexpected findings and continue to strengthen this field of intense research.

## Abbreviations

Rb: retinoblastoma; CDK: Cyclin-dependent kinase.

## Competing interests

The authors declare that they have no competing interests.

## Authors’ contributions

Both authors contributed equally to this manuscript and are listed in alphabetical order. Both authors read and approved the final manuscript.
